# Non-retrieval of inferior vena cava filters as a patient safety concern: evaluation of a new process improvement project to increase retrieval rates in a vascular and interventional radiology clinic

**DOI:** 10.1186/s13037-018-0151-7

**Published:** 2018-03-20

**Authors:** Joshua Brown, Jeffery Talbert, Ryan Pennington, Qiong Han, Driss Raissi

**Affiliations:** 1 Department of Pharmaceutical Outcomes and Policy, University of Florida College of Pharmacy, 1225 Center Drive HPNP #3320, Gainesville, FL 32610 USA; 2 Department of Pharmacy Practice and Science, University of Kentucky College of Pharmacy, Lexington, KY USA; 3 Department of Radiology, Division of Vascular and Interventional Radiology, University of Kentucky College of Medicine, Lexington, KY USA

**Keywords:** Inferior vena cava filters, Venous thromboembolism, Quality improvement, Retrieval

## Abstract

**Background:**

Retrieval of inferior vena cava filters (IVCFs) is important to decrease the long-term risk of complications associated with indwelling devices. Our hospital experienced low retrieval rates and implemented a low-cost intervention and evaluation for quality improvement. The working hypothesis was that a simple, mailed letter intervention could increase retrieval rates by increasing patient and primary care provider knowledge of the need for retrieval.

**Methods:**

For all prospective patients who received a retrievable IVCF during the intervention period from January 1, 2014 to February 29, 2016, patients and their primary care providers were mailed letters encouraging contact with the clinic for evaluation of eligibility for retrieval. The main outcome was retrieval of the IVCF if clinically indicated with a secondary outcome of time-to-retrieval. A pre-intervention control group from October 1, 2011 to December 31, 2013 was used to evaluate the impact of the intervention. Competing risks, time-to-event analysis was used to compare the pre- and post-intervention period retrieval rates correcting for patients who died during follow-up.

**Results:**

Between the pre- and post-intervention periods, crude retrieval rates increased from 4.4% to 8.1% with a 12-fold change at comparable time points. The time-to-retrieval in the pre-intervention period was a mean (SD) of 503 (207) days with a median (IQR) of 505 (301–742). In the post-intervention period, time-to-retrieval was a mean (SD) of 119 (83) days and with median (IQR) of 128 (38–164) days.

**Conclusions:**

This low-cost intervention significantly increased retrieval rates in a single clinic. However, retrieval rates remain low and can be further improved. Ongoing interventions, including improved patient follow-up and physician education, are being implemented to further improve retrieval and use of inferior vena cava filters. Implanting clinics should implement quality improvement initiatives to improve patient care and follow-up with IVCFs to ensure retrievals occur once clinically relevant in order to minimize long-term complications.

## Background

In the United States, inferior vena cava filters (IVCFs) are utilized in roughly 1-in-10 patients with venous thromboembolism (VTE) and only about one-third of these is ever retrieved [1, 2]. Recent medico-legal and regulatory attention into low retrieval rates of IVCFs elicited several institutional interventions aimed at improving retrieval in the patient population [3, 4]. This has been spurred by reports of overall low retrieval rates as well as FDA safety communications calling for increased and earlier retrieval of IVCFs once clinically indicated to improve patient outcomes – especially to prevent adverse events associated with indwelling devices [4].

In response to this attention, the Vascular and Interventional Radiology (VIR) clinic in the University of Kentucky Healthcare (UKHC) hospital instituted a retrospective review of retrieval rates along with a prospective letter mailing intervention to increase retrieval in patients for whom an IVCF is no longer indicated. We hypothesized that a simple, one time, mailed letter intervention could significantly impact retrieval rates while not interrupting clinical workflow. The design and impact of this intervention is described herein and implications for ongoing quality improvement research and initiatives are discussed.

## Methods

As part of a clinical practice improvement initiative, a registry of all IVCFs placed at the VIR clinic between October 1, 2011 and February 29, 2016 was created and housed within the Department of Radiology with a clinical study coordinator. The study coordinator retrospectively collected this database both before and after the intervention. Information collected included patient identifying information, referring physician, patient’s primary care physician, indication, and procedure details including date, and the retrieval date. The initiative and data collection were approved by the university Institutional Review Board.

Starting January 1, 2014, all patients with retrievable IVCFs implanted by VIR and their primary care or referring physicians were followed-up with a one-time letter sent within 3 months by the implanting physician and study coordinator regarding the need for eventual IVCF retrieval. In the letters, contact information to the VIR clinic was provided and contact was encouraged by either the primary care physician or patient. Other than the mailed letters, standard care was provided throughout the intervention period.

The primary outcome was retrieval of the IVCF with a secondary outcome of time-to-retrieval. A pre-intervention period between October 1, 2011 and December 31, 2013 was included for comparison and similarly included all patients. Retrieval rates and time-to-retrieval were compared between the pre- and post-intervention periods.

Patients were assumed to be eligible for retrieval until death was observed. The date of retrieval was noted, otherwise patients were censored at the beginning of the intervention period (for those in the pre-intervention group), or censored at the end of the data collection period (February 2016). Patients who died within 30 days of IVCF placement without having retrieval were excluded from the cohort. Death was considered a competing event in the calculation of the cumulative incidence of retrieval using the method by Fine and Gray to allow for comparison between the pre- and post-intervention groups. The mean, standard deviation (SD), median, and interquartile range (IQR) of the time-to-retrieval were also calculated.

## Results

A total of 184 and 93 IVCFs placed in the pre- and post-intervention periods. Of those, 10 and 3 patients were excluded from follow-up because they were deceased within 30 days after IVCF placement. Of the 90 patients in the intervention period, all patients were sent letters and 87 letters were sent to primary care providers as 3 patients did not have a provider noted in the medical record).

**Fig. 1:**
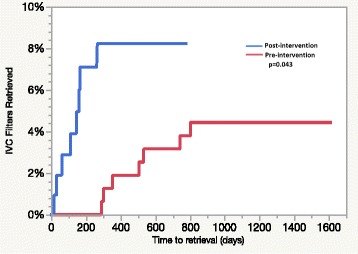
Cumulative incidence plot of time -to -retrieval after vena cava filter placement in the pre- and post-intervention periods

Of those in the pre-intervention period, 7/174 (4%) had their IVCF retrieved while 7/90 (7.8%) of those in the post-intervention period were retrieved. In the time-to-event analysis, which accounted for death during follow-up, the observed retrieval rate at a total of 802 days of follow-up prior to the letter intervention was 4.4% (Fig. 1). In the post-intervention period, the observed retrieval rate at 265 days of follow-up was 8.1% (Fig. 1). At an equivalent follow-up period with a cumulative incidence estimate available (288 days), the pre-intervention group’s estimated retrieval rate was < 1%, giving a relative rate of retrieval of 12.8 between the two comparable time periods. Overall, the cumulative incidence between the two groups was significantly different (*p* = 0.043). The time-to-retrieval in the pre-intervention period was a mean (SD) of 503 (207) days with a median (IQR) of 505 (301–742). In the post-intervention period, time-to-retrieval was a mean (SD) of 119 (83) days and with median (IQR) of 128 (38–164) days. Due to the minimal intervention implemented, minimal financial impact to the clinic was expected; thus, not evaluated.

## Discussion

This minimal intervention implemented in the VIR clinic resulted in an increase in the IVCF retrievals. Overall, the magnitude of this impact was on the order of a relative rate of 12.8 when limiting the analysis to an equivalent follow-up time of nearly 9 months. Likewise, there was a decrease in the time-to-retrieval, showing that retrievals were occurring at earlier intervals. This can be expected to decrease the risk of complications reported with longer indwell times such as IVCF fracture, migration, and IVC thrombosis.

Despite the large *relative* change in IVCF retrieval, the observed improved rate of 8.1% is among the lowest reported retrieval rates in the literature. Only one other known institution has reported a similar rate of 8.5% [5]. Due to this low rate, that institution also implemented an intervention that further increased their retrieval rate to over 50% [6].

The low rates in our clinic may be due to several factors. For one, the clinic is part of a level 1 trauma center serving a largely rural population, including the health disparate Appalachian region, making follow-up care difficult. The patient case-mix at UKHC is also known to be the most complex in the state, with higher comorbidity burden that correlates with the highest utilization of IVCFs [7, 8]. However, it should not be inferred that the hospital is dramatically different from other institutions where retrieval rates have been reported to be much higher than that observed in our institution [2]. Rather, it is likely there is selective reporting of retrieval rates in the literature as institutions may not be willing to disclose low retrieval due to concerns about the perception of quality at their institution. However, we recognize the need for transparency to increase accountability within the clinic and drive future improvement in care quality [9].

This intervention included only mailed letters and retrospective collection of patient data. Other interventions instituted at other hospitals have generally included common domains:

1. Patient and physician education regarding IVCFs [10, 11].
2. Communication regarding plans for retrieval at implantation or discharge planning [10].
  3.  A method of tracking patients (e.g. automated alerts through electronic medical records, patient registries, and/or active tracking) [6, 11]and
  4. An individual who takes responsibility for the entire process [12–14].

All of these various approaches alone or in combination showed improvements in retrieval rates. The addition of these workflows and responsibilities can result in newly generated clinic revenue, profiting from the increased patient follow up and billable clinical visits without a significant increase in workload [15]. Additionally, tracking of complications related to the presenting VTE indication, notation of existing transient or permanent contraindications to anticoagulation that justify continued use of IVCFs, standards for decision-making and communicating ongoing need for IVCF, and tracking of IVCF complications are needed for robust management of these patients. This retrospective study was limited in collection of this information which is important to assess the quality of care prior to and after IVCF implantation.

These concepts are currently being incorporated into a future intervention to further increase the retrieval rates in the UKHC VIR clinic. Moreover, this intervention is expanding to incorporate inter-departmental collaboration and communication in all implanting and referring specialties, not only VIR. Further interventions will focus not only on retrieval but also the judicious use of IVCFs by development of integrated clinical decision-support tools at the point of care along with continuing physician education [10].

## Conclusion

IVCF implanting clinics should seek to increase IVCF retrieval rates to decrease the long-term risk of device-related complications. In our institution, utilizing a minimal letter mailing intervention led to improved retrieval rates along with decreased the time-to-retrieval. However, given the post-intervention retrieval of only 8.1%, much more progress can be made to increase retrieval rates with a more thorough intervention. Similar simple interventions that are not costly in either man-power or cost can be universally implemented in implanting clinics to improve patient outcomes associated with IVCFs.

## Abbreviations

IQR: Interquartile range
IVCF: Inferior vena cava filter
SD: Standard deviations
UKHC: University of Kentucky Healthcare
VIR: Vascular and Interventional Radiology
VTE: Venous thromboembolism
